# Investigation of Protective Effects of Carvacrol and Hesperidin in Rats with Streptozotocin-Induced Diabetic Nephropathy

**DOI:** 10.3390/life16071067

**Published:** 2026-06-26

**Authors:** Nilüfer Kuruca, Tolga Güvenç

**Affiliations:** Faculty of Veterinary Medicine, Department of Pathology, Ondokuz Mayis University, 55189 Samsun, Türkiye; tguvenc@omu.edu.tr

**Keywords:** apoptosis, carvacrol, diabetes mellitus, diabetic nephropathy, hesperidin, streptozotocin

## Abstract

**Background:** Diabetes mellitus is a metabolic disorder characterized by chronic hyperglycemia resulting from impaired insulin secretion or action. Persistent hyperglycemia contributes to the development of micro- and macrovascular complications, particularly affecting the kidneys and leading to diabetic nephropathy through mechanisms involving oxidative stress, fibrosis and apoptosis. **Purpose:** This study aimed to investigate the protective effects of hesperidin and carvacrol, individually and in combination, on streptozotocin-induced diabetic nephropathy in a Type 1 diabetes rat model. **Study design:** An experimental in vivo animal study was conducted using a streptozotocin-induced Type 1 diabetic rat model. **Methods:** Sixty adult Wistar Albino rats were randomly divided into six groups (*n* = 10): Control, Diabetes, Vehicle, Diabetes + Carvacrol, Diabetes + Hesperidin, and Diabetes + Hesperidin + Carvacrol. Following a 6-week (42-day) experimental period, systemic necropsy was performed. Renal tissues were evaluated histopathologically using Periodic Acid–Schiff, Masson’s trichrome, and Best Carmine staining. Immunohistochemical analyses were conducted to assess Bcl-2, Bax, Caspase-3, Caspase-9 expression, and apoptotic cell death using the TUNEL assay. **Results:** Histopathological analysis demonstrated significant differences in tubular dilatation, intracellular vacuolization, and mesangial matrix expansion among groups (*p* < 0.05), whereas tubular atrophy, hyaline accumulation, mononuclear cell infiltration, and the number of sclerotic glomeruli were not significantly different (*p* > 0.05). Treatment with hesperidin and carvacrol significantly attenuated renal injury and fibrosis in diabetic rats (*p* < 0.001). Diabetic kidneys exhibited increased immunopositivity for Bax, Caspase-3, and Caspase-9 and decreased Bcl-2 expression (*p* < 0.001). Combined hesperidin and carvacrol treatment markedly reduced apoptotic cell numbers compared with the untreated diabetic group (*p* < 0.001). **Conclusions:** The combined administration of hesperidin and carvacrol exerts protective effects against diabetic nephropathy, potentially through modulation of the Bcl-2/Bax-mediated apoptotic pathway and attenuation of renal fibrosis.

## 1. Introduction

Diabetes Mellitus (DM) is a chronic carbohydrate metabolism disorder that occurs when the production of or responsiveness to insulin, which is responsible for regulating blood glucose levels, is impaired. Although insulin therapy is initiated in individuals diagnosed with diabetes, numerous complications still arise in diabetic patients experiencing wide fluctuations in blood glucose. These irregularities in insulin levels lead to a cascade of detrimental outcomes across various metabolic processes [1]. Persistent hyperglycemia can result in microvascular complications such as retinal, neural, and renal damage, as well as macrovascular complications including atherosclerosis, myocardial infarction, stroke, and diabetic coma [2]. Diabetic nephropathy (DN), one of the most prevalent complications of diabetes, seriously threatens renal function in diabetic individuals, occurring in 20–40% of patients diagnosed with DM [3]. High blood glucose levels play a major role in the progression of renal dysfunction and the exacerbation of DN lesions. The pathogenesis of DN is not yet fully understood; however, it characteristically progresses through five distinct stages. The initial stage involves hyperfiltration driven by hyperglycemia. The second stage may present histological alterations that are not yet associated with clinical findings. The third stage is characterized as incipient nephropathy, where albumin is excreted in the urine. Overt proteinuria occurs in the fourth stage, while the fifth and final stage is end-stage renal disease (ESRD), which necessitates renal replacement therapy or kidney transplantation. Current therapeutic approaches for patients with DN focus on regulating blood glucose and blood pressure levels. Although managing hypertension and blood glucose with drugs that modulate the renin–angiotensin system (RAS) slows the progression rate of diabetic kidney disease (DKD), these interventions remain insufficient to fully prevent DN complications [4]. Consequently, if necessary precautions are not taken and novel treatments are not developed, DN is poised to become the leading cause of kidney failure in the coming years.

Many traditional plants are utilized as effective, reliable, and economical remedies for the treatment of diabetes and the prevention of its complications. The use of herbal preparations to manage diabetes complications is highly prevalent, particularly in underdeveloped and developing countries [5]. Since synthetic agents often present numerous adverse side effects on human health, recent research has increasingly focused on herbal nutrition and natural compounds to control diabetes-related complications [6,7]. Carvacrol (CAR; 2-methyl-5-isopropylphenol) is a monoterpene phenolic agent predominantly found in the essential oils of various plant species (such as marjoram, mugwort, and thyme) belonging to the Lamiaceae family [8,9,10]. CAR, which is also widely used as a food additive, possesses a broad spectrum of biological and pharmacological properties, including antioxidant, antidiabetic, antibacterial, antifungal, anticarcinogenic, antiapoptotic, anti-inflammatory, and hepatoprotective activities [8,11,12]. The potent antioxidant capacity of CAR, demonstrated in both in vitro and in vivo studies, is directly attributed to the presence of the hydroxyl group attached to its aromatic ring [13,14]. Similarly, citrus fruits, which are rich in nutrients and bioactive components, play an important role in the prevention and management of obesity and metabolic disorders. Hesperidin (HP; 3′,5,7-trihydroxy-4′-methoxyflavanone-7-rhamnoglucoside) is one of the major bioflavonoid compounds found in citrus fruits [15,16]. Extensive analyses have been conducted to define the pharmacological effects of HP [17], and toxicological evaluations have confirmed that it exhibits no significant adverse side effects [18,19]. Studies have revealed that HP exerts anti-inflammatory, antihyperglycemic [20,21,22], antihyperlipidemic [22,23], anticarcinogenic, antioxidative [24], antifungal, antiviral, immunomodulatory, hepatoprotective, cardioprotective, antidepressant, and neuroprotective properties in diabetic models [25]. Furthermore, HP has been reported to inhibit Nuclear Factor Kappa B (NF-κB) and oxidative stress in the liver and kidneys of diabetic animals [26], reduce serum levels of proinflammatory cytokines and oxidative stress markers [27], protect against high-glucose-induced oxidative stress and apoptosis in retinal ganglion cells [28], and preserve pancreatic β cells morphology and function by suppressing oxidative and endoplasmic reticulum (ER) stress [29].

The present study investigated the potential protective effects of hesperidin (HP) and carvacrol (CAR), two natural compounds renowned for their antioxidant properties, in a streptozotocin-induced diabetic nephropathy rat model. Histopathological, immunohistochemical, and TUNEL analyses were performed to comprehensively evaluate renal injury and apoptosis. In addition, the effects of HP and CAR, administered individually or in combination, on apoptosis-related proteins and diabetic nephropathy-associated structural renal alterations were assessed.

## 2. Materials and Methods

### 2.1. Animals and Ethical Permissions

Sixty adult male Wistar Albino rats (6–8 weeks old, weighing 160–200 g) were obtained from the Ondokuz Mayis University Experimental Animal Research Center (Samsun, Türkiye). The rats were randomly allocated into six experimental groups (*n* = 10): Control, Diabetes, Vehicle, Diabetes + Hesperidin, Diabetes + Carvacrol, and Diabetes + Hesperidin + Carvacrol. All animals were housed under controlled environmental conditions (temperature: 22 ± 2 °C, humidity: 55% ± 5%) with a 12 h light/dark cycle and were provided standard laboratory chow and water ad libitum. All experimental procedures were conducted in strict accordance with the Guide for the Care and Use of Laboratory Animals published by the National Institutes of Health (NIH) and were approved by the Institutional Animal Care and Use Committee of Ondokuz Mayis University (Approval No: 2020-13). The sample size was determined by an a priori power analysis using G*Power software (v3.1.9.7). Assuming a significance level (α) of 0.05 and a statistical power of 0.95, the analysis indicated that 10 animals per group would be sufficient to detect biologically relevant differences among the experimental groups.

### 2.2. Hesperidin and Carvacrol Supplementation and Induction of the Diabetes Model

Hesperidin (Sigma-Aldrich, St. Louis, MO, USA, Cat. No. H5254-100G) and carvacrol (Sigma-Aldrich, St. Louis, MO, USA, Cat. No. W224511, Natural, 99% purity) were obtained commercially. Carvacrol (75 mg/kg) was dissolved in olive oil, and hesperidin (50 mg/kg) was dissolved in distilled water. Both compounds were administered via orogastric gavage daily at volumes adjusted to individual body weights, not exceeding 1 mL per animal. The vehicle group received olive oil alone by orogastric gavage in volumes equivalent to those used for carvacrol administration. Olive oil was used as the vehicle because it served as the solvent for carvacrol. Inclusion of the vehicle group allowed evaluation of any effects related to the vehicle itself.

Type 1 diabetes mellitus was induced via a single intraperitoneal (IP) injection of freshly prepared streptozotocin (STZ; Sigma-Aldrich, Cat. No. S0130-1G) at a dose of 50 mg/kg dissolved in 0.1 M citrate buffer (pH 4.5). Seventy-two hours post-STZ injection, blood samples were obtained from the tail vein of overnight-fasted rats. Blood glucose concentrations were measured using an *On Call Plus* (ACON Laboratories, Inc., San Diego, CA, USA) glucometer. Rats with blood glucose levels exceeding 250 mg/dL were confirmed as diabetic and included in the study. Animals failing to develop diabetes after the initial injection received a second STZ dose of 50 mg/kg. To prevent initial fatal hypoglycemia induced by massive insulin release following STZ administration, rats were provided with saturated sugar water for the first 24 h.

At the end of the 42-day experimental period, rats were anesthetized with an IP injection of a ketamine (100 mg/kg) and xylazine (10 mg/kg) mixture. Blood samples were collected via cardiac puncture into vacuum gel tubes, and the animals were immediately euthanized by decapitation for tissue harvesting.

### 2.3. Histopathology and Immunohistochemistry

Kidney samples were fixed in 10% buffered formalin, dehydrated through a graded ethanol series, cleared in xylene, and embedded in paraffin. Sections were cut at a thickness of 5 μm and stained with Periodic Acid–Schiff (PAS), Masson’s trichrome, and Best’s Carmine according to standard protocols. Histopathological examinations and photomicrography were performed using a light microscope (Nikon Eclipse E600, Nikon Corporation, Tokyo, Japan) equipped with a digital imaging system (Nikon DS Camera Head DS-5M, Nikon Corporation, Tokyo, Japan).

For immunohistochemical evaluations, the streptavidin–biotin peroxidase complex (SABC) technique was performed using a commercial detection kit (Thermo Scientific, Ultravision Large Volume Detection System, Fremont, CA, USA) according to the manufacturer’s instructions. Antigen retrieval was performed by heating the sections in a citrate buffer (pH 6.0) solution in a microwave oven at 600 W for 20 min. Endogenous peroxidase activity was blocked by incubating the slides in 0.5% hydrogen peroxide (H_2_O_2_) in methanol for 10 min. Following a protein blocking step, sections were incubated overnight at 4 °C with the following primary antibodies: Bcl-2 (Novus Biologicals, Centennial, CO, USA; NB100-56098, 1:100 dilution), Bax (Novus Biologicals, Centennial, CO, USA; NB100-56095, 1:100 dilution), active/cleaved Caspase-3 (Novus Biologicals, Centennial, CO, USA; NB100-56113, 1:150 dilution), and Caspase-9 (Novus Biologicals, Centennial, CO, USA; NB100-56118, 1:150 dilution). After rinsing with PBS, the sections were incubated with the SABC reagent for 1 h at room temperature. Immunoreactivity was visualized using a 3-amino-9-ethylcarbazole (AEC) chromogen kit (Abcam, Cambridge, UK; ab64252, ready-to-use). The sections were counterstained with Mayer’s hematoxylin and mounted using an aqueous mounting medium (Sigma-Aldrich, C9368). Ten random fields from both the cortical and medullary regions of each kidney section were photographed at 200× magnification. Quantitative image analysis was conducted using the Bs200Pro Image Analysis System (BAB Digital Imaging System, 2007, Ankara, Turkey), and the expression levels were evaluated based on the calculated percentages of positively stained areas.

### 2.4. TUNEL (Terminal Deoxynucleotidyl Transferase dUTP Nick-End Labeling) Assay

Apoptotic cell death in renal tissues was evaluated using a commercial TUNEL Apoptosis Detection Kit (HRP–DAB) (Elabscience, Houston, TX, USA, Cat. No. E-CK-A331) according to the manufacturer’s protocol. Briefly, paraffin sections were deparaffinized, rehydrated, treated with Proteinase K for antigen retrieval, and incubated with 3% H_2_O_2_ to block endogenous peroxidase activity. The sections were then incubated with the terminal deoxynucleotidyl transferase (TdT) labeling solution, followed by incubation with Streptavidin-HRP. Chromogenic visualization was performed using 3,3′-diaminobenzidine (DAB), which labels apoptotic nuclei brown. The sections were counterstained with Mayer’s hematoxylin (rendering non-apoptotic nuclei blue), dehydrated, cleared in xylene, and mounted with Entellan (Merck, KGaA, Darmstadt, Germany, Cat. No. 107961). Apoptotic cells were quantified at 200× magnification across 20 randomly selected fields per animal (10 cortical and 10 medullary fields). A total of 1000 cells were counted per region, and any distinct brown-stained nucleus was recorded as a TUNEL-positive cell to determine the apoptotic index.

### 2.5. Statistical Analysis

Statistical analyses were performed using SPSS software (version 21.0; IBM Corp., Armonk, NY, USA). Data normality was assessed using skewness–kurtosis values and the Shapiro–Wilk test, while homogeneity of variances was evaluated using Levene’s test. Normally distributed parametric data were analyzed using one-way analysis of variance (ANOVA) followed by Duncan’s post hoc test for multiple comparisons. Non-normally distributed non-parametric data were analyzed using the Kruskal–Wallis H test, followed by the Mann–Whitney U test for pairwise comparisons where appropriate. Parametric results are expressed as mean ± standard deviation (SD), and non-parametric data are presented as median (minimum–maximum). Statistical significance was defined as *p* ≤ 0.05.

## 3. Results

### 3.1. Body Weight Changes

The weekly body weight changes in the rats throughout the 6-week experimental period are presented in Table 1. The control and vehicle groups exhibited a continuous and significant increase in body weight from day 1 until necropsy (*p* < 0.05). Conversely, the diabetes group demonstrated a severely restricted weight gain trajectory compared to the healthy controls, remaining significantly lower at the end of the study (*p* < 0.05). Administering either carvacrol (D + CAR) or hesperidin (D + HP) individually failed to reverse diabetes-induced growth suppression. The D + HP group showed a progressive weight loss during the first three weeks, followed by a limited recovery that remained significantly lower than the baseline value by week 6. Similarly, the D + CAR group exhibited stagnant body weight profiles throughout the experimental timeline. Notably, the combined treatment (D + HP + CAR) significantly mitigated diabetes-induced weight restriction, resulting in a significantly higher final body weight compared to both the untreated diabetes group and the groups treated with each compound alone (*p* < 0.05).

### 3.2. Metabolic Cage Results

Metabolic cage parameters, including feed and water consumption alongside feces and urine outputs measured 24 h before necropsy, are depicted in Figure 1. Standard STZ-induced diabetic alterations were clearly evident, with the diabetes (D) group exhibiting significant increases in all metabolic parameters compared to the control (C) and vehicle (V) groups (*p* < 0.05). Regarding water consumption and urine volume, the C and V groups showed baseline levels that were statistically identical (*p* > 0.05). All diabetes-induced groups (D, D + CAR, D + HP, and D + HP + CAR) displayed a marked, significant elevation in both fluid intake and urine output compared to the controls (*p* < 0.05). No statistically significant differences were observed among the diabetic treatment groups. Feed consumption was significantly higher in all diabetic groups (D, D + CAR, D + HP, and D + HP + CAR) than in the V group (*p* < 0.05), while the control group (C) showed no statistical difference from either side (*p* > 0.05). Fecal output directly matched this feed consumption pattern; the D, D + CAR, and D + HP + CAR groups produced significantly more feces than both healthy groups (C and V) (*p* < 0.05). The D + HP group remained at an intermediate level, showing no significant difference from the other groups (*p* > 0.05).

### 3.3. Blood Glucose Levels

Postprandial blood glucose concentrations measured at the end of the 6-week experimental period differed significantly among the groups (*p* < 0.001; Figure 2). The control (C) group exhibited the lowest glucose levels, whereas all STZ-treated groups developed marked hyperglycemia. The highest glucose concentrations were observed in the Diabetes + Hesperidin (D + HP) group, which did not differ significantly from the Diabetes (D) and Diabetes + Carvacrol (D + CAR) groups. In contrast, the Diabetes + Hesperidin + Carvacrol (D + HP + CAR) group showed significantly lower glucose levels than the D + HP group (*p* < 0.05). Although glucose concentrations in the D + HP + CAR group were lower than those in the diabetic group, this difference was not statistically significant.

### 3.4. Renal Findings

Gross morphometric measurements of the right kidney are summarized in Table 2. Total kidney length (*p* = 0.059), total kidney width (*p* = 0.999), and medullary thickness (*p* = 0.149) did not differ significantly among the experimental groups. In contrast, kidney weight differed significantly among the groups (*p* = 0.001). The D + HP + CAR group exhibited the highest kidney weight (1.361 ± 0.16 g), which was significantly greater than those of the other groups (*p* < 0.05). The lowest kidney weight was observed in the D + HP group (1.111 ± 0.14 g), which differed significantly from the control (C) and vehicle (V) groups (*p* < 0.05). No significant differences were detected among the D, D + CAR, and D + HP groups (*p* > 0.05). Cortical thickness also differed significantly among the groups (*p* = 0.047). The D + HP group exhibited the greatest cortical thickness (2.37 ± 0.36 mm), which was significantly higher than those of the control and D + HP + CAR groups (*p* < 0.05). In contrast, cortical thickness in the D + HP + CAR group (1.90 ± 0.34 mm) did not differ significantly from that of the control group (1.89 ± 0.31 mm; *p* > 0.05).

Gross morphometric measurements of the left kidney are summarized in Table 3. Total kidney length (*p* = 0.338), width (*p* = 0.652), cortical thickness (*p* = 0.189), and medullary thickness (*p* = 0.138) did not differ significantly among the experimental groups. In contrast, left kidney weight differed significantly among the groups (*p* = 0.001). The lowest kidney weight was observed in the diabetes (D) group (1.089 ± 0.14 g), which was significantly lower than that of the control (C) group (1.330 ± 0.23 g; *p* < 0.05). The D + CAR and D + HP groups showed kidney weights that did not differ significantly from the diabetic group (*p* > 0.05). In contrast, the D + HP + CAR group exhibited a kidney weight of 1.295 ± 0.15 g, which did not differ significantly from those of the control and vehicle (V) groups (*p* > 0.05).

Relative kidney weights, calculated by normalizing kidney weight to body weight, are presented in Table 4. Significant differences were observed among the experimental groups for both the right and left kidneys (*p* < 0.05). For the relative right kidney weight, the D + CAR group exhibited the highest values among all groups. The control (C) and vehicle (V) groups showed similar relative kidney weights and both differed significantly from the diabetic and treatment groups (*p* < 0.05). A similar pattern was observed for the relative left kidney weight. The control and vehicle groups exhibited significantly lower values than the diabetic and treatment groups (*p* < 0.05). No significant differences were detected among the D, D + CAR, D + HP, and D + HP + CAR groups (*p* > 0.05).

### 3.5. Histopathological Findings

#### 3.5.1. Periodic Acid–Schiff (PAS) Staining

Renal histomorphometric measurements are summarized in Table 5. Bowman’s space width (*p* = 0.185) and the visceral layer thickness of Bowman’s capsule (BKv; *p* = 0.247) did not differ significantly among the experimental groups. In contrast, glomerular size differed significantly among the groups (*p* = 0.017). The diabetes (D) group exhibited the largest glomerular size (104.04 ± 7.3 µm), which was significantly greater than those of the vehicle (V), D + CAR, D + HP, and D + HP + CAR groups (*p* < 0.05). No significant differences were observed between the control (C) group and the treatment groups (*p* > 0.05). The parietal layer thickness of Bowman’s capsule (BKp) also differed significantly among the groups (*p* = 0.001). The control group exhibited the greatest BKp thickness (1.79 ± 0.2 µm), whereas significantly lower values were observed in the D, V, D + CAR, D + HP, and D + HP + CAR groups (*p* < 0.05). Tubular basement membrane (TBM) thickness differed significantly among the groups (*p* = 0.03). TBM thickness was significantly greater in the C and D + CAR groups than in the D + HP group (*p* < 0.05).

Semi-quantitative histopathological evaluation revealed significant differences in tubular dilatation, intracellular vacuolization, and mesangial matrix expansion (*p* < 0.05; Table 6, Figure 3). Tubular dilatation scores were significantly higher in the D group than in the C and V groups (*p* < 0.05). Mesangial matrix expansion also differed significantly among the groups (*p* < 0.001), with the highest scores observed in the D group. The D + HP and D + HP + CAR groups exhibited lower mesangial matrix expansion scores than the D group, although values remained higher than those observed in the control group.

#### 3.5.2. Masson’s Trichrome Staining

Quantitative analysis of renal collagen deposition, assessed by Masson’s trichrome staining, revealed significant differences in fibrotic area percentages among the experimental groups (*p* < 0.05; Figure 4). The diabetes (D) group exhibited the highest fibrotic area percentage (7.54 ± 2.42%, a). Fibrotic area percentages in the D + CAR (b) and D + HP (c) groups were significantly lower than that of the D group (*p* < 0.05). The D + HP + CAR group exhibited the lowest fibrotic area percentage (2.73 ± 0.86%, e), which was significantly lower than those of the D, D + CAR, and D + HP groups (*p* < 0.05). No significant difference was observed between the D + HP + CAR and vehicle (V) groups, whereas the D + HP + CAR group differed significantly from the control (C) group, as indicated by the superscript letter designations.

Histopathological examination of Masson’s trichrome-stained renal sections supported the quantitative fibrosis findings. The control (Figure 5A) and vehicle (Figure 5C) groups exhibited normal renal histological architecture, with collagen staining primarily confined to glomerular basement membranes and the peritubular interstitium (black arrows). In the diabetes (D) group (Figure 5B), increased collagen deposition and mesangial matrix expansion were observed within the glomeruli and interstitial areas (black arrowheads). The D + CAR group (Figure 5D) exhibited collagen deposition and mesangial matrix expansion, whereas the D + HP group (Figure 5E) showed less extensive collagen staining than the diabetic group. Among the treatment groups, the D + HP + CAR group (Figure 5F) exhibited the lowest degree of collagen deposition and mesangial matrix expansion. These histopathological findings were consistent with the quantitative fibrosis measurements presented in Figure 4.

#### 3.5.3. Best’s Carmine Staining

Histochemical evaluation using Best’s carmine staining was performed to assess glycogen accumulation in renal tissues (Figure 6 and Figure 7). No glycogen deposition was observed in the glomeruli of any experimental group. In contrast, glycogen accumulation was detected in the renal tubular epithelium. The diabetes (D) group exhibited intense carmine-positive staining within tubular epithelial cells, indicating increased glycogen deposition. The control (C) and vehicle (V) groups showed no detectable carmine-positive staining. The D + HP + CAR group exhibited no detectable tubular glycogen accumulation, whereas minimal focal carmine-positive staining was observed in the D + CAR and D + HP groups.

### 3.6. Immunohistochemical Findings

Bcl-2 immunopositivity in both the cortex and medulla was lowest in the diabetes group. The highest positivity occurred in the diabetes + hesperidin + carvacrol group. The diabetes+ hesperidin and diabetes + carvacrol groups also showed higher Bcl-2 levels than the diabetes group (*p* < 0.001) (Figure 8). All groups showed specific Bcl-2 immunoreactivity. Strong tubular cytoplasmic and nuclear staining was present in the diabetes + hesperidin + carvacrol, diabetes + carvacrol, and diabetes + hesperidin groups, whereas the diabetic group showed only weak cytoplasmic staining (Figure 9 and Figure 10).

Bax immunopositivity in both the cortex and medulla was highest in the diabetes group. The diabetes + hesperidin + carvacrol group showed the lowest levels, with significant reductions. The diabetes + carvacrol and diabetes + hesperidin groups exhibited cortical Bax levels similar to the control and vehicle groups. In the medulla, Bax positivity in the diabetes + carvacrol group was lower than in the diabetes group but not statistically significant, while the diabetes+ hesperidin group showed reduced levels comparable to the control group (Figure 11, Figure 12 and Figure 13).

Active Caspase-3 levels differed significantly among the groups (*p* < 0.01). The diabetes group showed the highest cortical and medullary positivity (*p* < 0.001). The diabetes + carvacrol, diabetes + hesperidin, and diabetes + hesperidin + carvacrol groups had lower Caspase-3 positivity than the diabetes group but higher levels than the control and vehicle groups (Figure 14, Figure 15 and Figure 16).

Significant differences in Caspase-9 immunopositivity were observed among the groups (*p* < 0.01). The diabetes group showed the highest cortical and medullary positivity (*p* < 0.001). The diabetes + carvacrol, diabetes + hesperidin, and diabetes + hesperidin + carvacrol groups exhibited lower positivity than the diabetes group, with varying levels between the control, vehicle, and combined-treatment groups. In the medulla, all treatment groups showed reduced positivity compared with the diabetes group but higher levels than the control group (Figure 17, Figure 18 and Figure 19).

### 3.7. TUNEL Findings

Quantitative evaluation of apoptosis using the TUNEL assay revealed significant differences in TUNEL-positive cell counts among the experimental groups in both the renal cortex (*p* < 0.001) and medulla (*p* < 0.001). In the cortical region, the diabetes (D) group exhibited significantly higher numbers of TUNEL-positive cells than the control (C) and vehicle (V) groups. The D + CAR group showed significantly lower cortical apoptotic cell counts than the diabetic group. Cortical TUNEL-positive cell counts in the D + HP and D + HP + CAR groups were also significantly lower than those observed in the D group. Among the diabetic groups, the D + HP + CAR group exhibited the lowest number of cortical TUNEL-positive cells. A similar pattern was observed in the renal medulla. The D group exhibited the highest number of TUNEL-positive cells, whereas the D + CAR, D + HP, and D + HP + CAR groups showed significantly lower apoptotic cell counts than the diabetic group. The D + HP and D + HP + CAR groups exhibited the lowest medullary TUNEL-positive cell counts among the diabetic groups (Figure 20, Figure 21 and Figure 22).

## 4. Discussion

Diabetes mellitus is a metabolic disorder characterized by hyperglycemia resulting from inadequate insulin secretion or insulin resistance, leading to progressive damage in multiple organs [30,31]. Its incidence is rising globally, and it is now among the leading causes of mortality alongside cancer and cardiovascular diseases [32]. Chronic hyperglycemia is known to cause structural and functional impairment in tissues such as the eyes, kidneys, heart, blood vessels, and nerves [33,34]. Diabetic nephropathy (DN), affecting at least 30% of diabetic patients, presents a major public health concern [35]. Due to the complex pathogenesis of DN, effective therapeutic options are limited, highlighting the need for new agents. Natural products have shown promising protective effects against DN development [36].

Diabetic rats typically show polyphagia, polydipsia, and significant weight loss due to impaired carbohydrate utilization, enhanced lipid catabolism, and excessive protein degradation [37,38,39]. In this study, body weight was monitored for six weeks, and all groups except the diabetic + hesperidin group gained weight. Weight gain was lower in the diabetic group than in controls, and even more limited in the diabetic + hesperidin and diabetic + carvacrol groups. Only the combined hesperidin + carvacrol treatment showed a more favorable body weight profile than the individual treatment groups, suggesting that the combined administration was more effective than either compound alone.

STZ-induced diabetic rats typically display polyuria, polydipsia, and weight loss [40,41]. In this study, diabetic rats consumed about 3.5 times more water than controls, while the hesperidin, carvacrol, and combined-treatment groups drank less water than the diabetic group but produced more urine. Hyperglycemia was elevated in the diabetic group compared with controls. Hesperidin or carvacrol alone did not significantly reduce blood glucose levels, whereas their combined administration resulted in a significant decrease. Although the exact mechanism underlying this effect was not investigated in the present study, it may be related to the complementary biological activities of the two compounds. Hesperidin has been reported to exert anti-inflammatory and vasoprotective effects, whereas carvacrol possesses potent antioxidant and antiapoptotic properties. Therefore, the combined treatment may provide greater metabolic benefits than either compound alone.

Hyperglycemia in diabetes contributes to renal hypertrophy and the development of diabetic nephropathy [42,43]. While some studies report increased kidney weight in diabetic rats due to hypertrophy [44,45], others have found no difference [46]. In the present study, relative kidney weights were higher in the diabetic group than in controls, whereas the hesperidin, carvacrol, and combined treatment groups showed values similar to the diabetic group. These findings suggest that the treatments had limited effects on relative kidney weight. Hyperglycemia promotes renal fibrosis by increasing profibrotic factor expression [47], and diabetes is known to enhance fibrotic changes in the kidney [48]. In this study, fibrosis was higher in diabetic rats than in controls, while hesperidin and carvacrol reduced fibrosis despite not lowering glucose levels. This antifibrotic activity aligns with data showing that carvacrol decreases serum urea and creatinine levels, activates the Nrf-2 antioxidant axis, and directly downregulates key fibrosis-related genes including COL1A1 and COL3A1 [49]. Furthermore, carvacrol has been reported to modulate apoptosis-related pathways through restoration of the Bax/Bcl-2 balance [49], which may contribute to the reduced fibrosis and apoptosis observed in the present study.

The histopathological findings suggested that diabetic nephropathy was at an early to intermediate stage, likely reflecting the relatively short 6-week experimental period. The diabetic group exhibited increased glomerular size, mesangial matrix expansion, and tubular vacuolization, consistent with previously reported diabetic renal lesions [50,51]. Treatment with hesperidin and carvacrol was associated with reduced tubular degeneration and mesangial matrix expansion compared with the diabetic group. Best’s Carmine staining demonstrated glycogen accumulation within renal tubular epithelial cells, a finding commonly associated with severe hyperglycemia and diabetic renal injury [52,53].

Acute and chronic hyperglycemia induce oxidative stress, leading to apoptosis in tubular and glomerular cells, as demonstrated in both animal models and in vitro systems [54,55,56]. Hyperglycemia has been shown to promote ROS-dependent mesangial cell apoptosis through Bax-mediated mitochondrial permeability and cytochrome c release [57]. High glucose increases the Bax/Bcl-2 ratio and activates caspase-3 via mitochondrial cytochrome c release in murine and human mesangial cells [58]. In renal tubular epithelial cells, hyperglycemia decreases Bcl-2 expression while upregulating Bax [59], consistent with earlier reports showing increased pro-apoptotic Bax and reduced anti-apoptotic Bcl-2/Bcl-XL expression in diabetes [60]. Caspase-3 and caspase-9 are key mediators of high-glucose-induced apoptosis in proximal tubular cells [61]. In the present study, STZ-induced diabetes increased renal apoptosis, consistent with previous experimental findings [62]. Recent evidence has further highlighted the central role of programmed cell death pathways in the progression of diabetic kidney disease. Liu et al. (2024) reported that chronic hyperglycemia promotes mitochondrial dysfunction, oxidative stress, and activation of intrinsic apoptotic pathways, resulting in increased Bax expression and activation of Caspase-9 and Caspase-3, accompanied by suppression of Bcl-2. These alterations contribute to tubular injury, glomerular dysfunction, and progressive renal fibrosis. Therefore, modulation of the Bax/Bcl-2/Caspase signaling axis is currently considered a promising therapeutic strategy for slowing the progression of diabetic nephropathy [63]. These observations further support the current understanding that apoptosis is not merely a consequence of diabetic nephropathy but also an important driver of disease progression.

Immunohistochemical analysis revealed that Bcl-2 immunoreactivity tended to be lower in the diabetic group compared with the control group, although this difference did not reach statistical significance. In contrast, all treatment groups exhibited higher Bcl-2 expression than the diabetic group. Bax immunopositivity was highest in the diabetic group and lowest in the combined hesperidin + carvacrol group. These findings are supported by data demonstrating that hesperetin increases the viability of human renal tubular cells (HK-2) under high-glucose conditions and blocks apoptosis by suppressing oxidative stress [64]. Hesperidin has been reported to downregulate pro-apoptotic markers such as Bax and Caspase-3 while upregulating the anti-apoptotic protein Bcl-2 in renal tissues [65], thereby exerting protective effects against STZ-induced renal injury. Furthermore, this flavonoid attenuates the TGF-β fibrosis signaling pathway, thereby preserving overall renal function under diabetic stress [64]. Collectively, these findings are consistent with the reduced Bax expression and increased Bcl-2 immunoreactivity observed in the treatment groups in the present study.

The TUNEL method is widely used to detect apoptosis by labeling DNA strand breaks in situ. During apoptosis, endonucleases cleave genomic DNA, generating 3′-OH ends that can be labeled with biotin-dUTP by terminal deoxynucleotidyl transferase (TdT), followed by streptavidin–HRP binding and DAB visualization. Although useful, TUNEL staining may mark non-apoptotic cells under certain conditions such as active transcription or necrosis, raising concerns about specificity [66,67,68,69]. High glucose has been shown to induce apoptosis in renal tubular, glomerular, and interstitial cells in rats [70], and STZ-induced diabetes increases TUNEL-positive cells particularly in tubular compartments, but not in glomeruli [45,71]. In this study, the highest number of TUNEL-positive cells in both cortex and medulla was observed in the diabetic group, mainly in proximal, distal, collecting tubules, and Henle’s loop. The lowest counts occurred in the hesperidin + carvacrol group. Combining hesperidin, which exerts anti-inflammatory and vasoprotective effects, with carvacrol, which possesses potent antioxidant and antiapoptotic properties, may provide complementary protection against diabetic renal injury. The superior effects observed in the combined treatment group compared with the individual treatment groups support the possibility of a synergistic interaction between these compounds. This observation is consistent with recent evidence demonstrating that simultaneous attenuation of oxidative stress, inflammation, fibrosis, and apoptosis can provide greater renoprotective effects than targeting a single pathogenic pathway alone [49,63,65]. The lower number of TUNEL-positive cells observed in the combined treatment group may therefore be associated with coordinated modulation of apoptosis-related pathways involving Bcl-2, Bax, Caspase-9, and Caspase-3.

Although the present findings are promising, their clinical relevance should be interpreted with caution. The beneficial effects observed in rats may not directly translate to humans because differences in absorption, bioavailability, metabolism, and dosing can influence treatment outcomes. Previous studies have reported favorable safety profiles for both hesperidin and carvacrol; however, their long-term efficacy and safety in diabetic patients remain unclear. Similar renoprotective effects have also been described for other natural compounds, such as curcumin, resveratrol, and quercetin, which act through antioxidant and anti-inflammatory mechanisms. Therefore, hesperidin and carvacrol may represent potential adjunctive therapeutic agents for diabetic nephropathy, although further studies are required before clinical application can be considered.

To the best of our knowledge, this is the first study to examine the effects of hesperidin and carvacrol on apoptosis-related proteins (Bcl-2, Bax, caspase-3, and caspase-9) in an STZ-induced type 1 diabetic nephropathy model. Previous research has shown that hyperglycemia alters apoptotic protein expression and contributes to renal damage. Taken together, the present findings suggest that hesperidin and carvacrol exert renoprotective effects through mechanisms involving attenuation of fibrosis and apoptosis in addition to their metabolic actions. The superior efficacy observed following combined administration further supports the potential benefit of simultaneously targeting multiple pathogenic pathways involved in diabetic nephropathy.

Several limitations of the present study should be acknowledged. Long-term glycemic control was assessed only by periodic blood glucose measurements, as HbA1c determination and continuous glucose monitoring were not performed. In addition, molecular analyses such as Western blotting, qPCR, or ELISA were not conducted. Therefore, the findings regarding Bcl-2, Bax, Caspase-3, and Caspase-9 are based on immunohistochemical evaluation. Furthermore, the six-week experimental period may not fully reflect the late-stage progression of diabetic nephropathy. Finally, since streptozotocin-induced diabetes primarily represents a Type 1 diabetes model, caution should be exercised when extrapolating these findings to Type 2 diabetes-associated nephropathy. Future studies incorporating molecular approaches, comprehensive glycemic assessments, longer experimental durations, and Type 2 diabetes models are warranted to further elucidate the mechanisms underlying the renoprotective effects of hesperidin and carvacrol and to validate their potential therapeutic applicability.

## 5. Conclusions

Diabetes mellitus is a global endocrine disorder with increasing incidence driven by lifestyle changes and obesity. While conventional antidiabetic therapies are widely used, interest in plant-derived compounds has grown due to their potential therapeutic effects and lower toxicity profiles. This study demonstrates that hesperidin and carvacrol, particularly when combined, exert renoprotective effects by reducing apoptosis in an STZ-induced diabetic nephropathy model. The findings suggest that the combined administration of hesperidin and carvacrol may attenuate renal injury through antioxidant, anti-inflammatory, and antiapoptotic mechanisms. Further molecular and mechanistic studies are required to clarify the precise pathways underlying these protective effects.

## Figures and Tables

**Figure 1:**
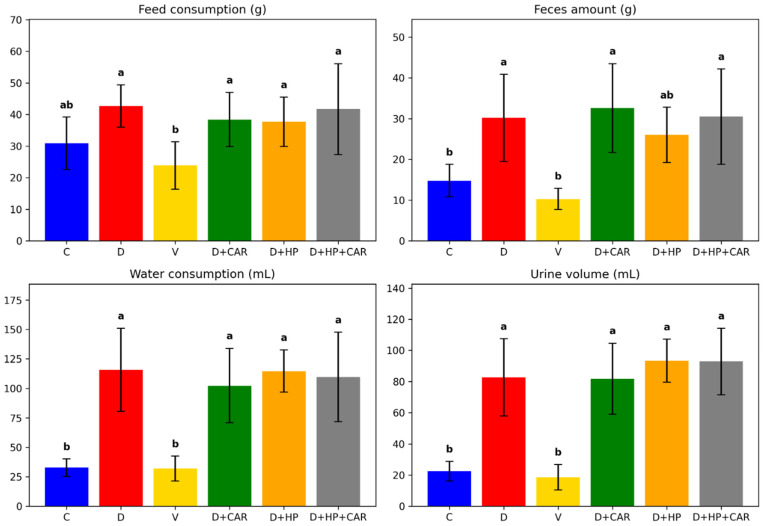
Feed consumption (g), feces amount (g), water consumption (mL), and urine volume (mL) in the Control (C), Diabetic (D), Vehicle (V), Diabetic + Carvacrol (D + CAR), Diabetic + Hesperidin (D + HP), and Diabetic + Hesperidin + Carvacrol (D + HP + CAR) groups. Data are presented as mean ± standard deviation (SD). Different letters above the bars indicate statistically significant differences among groups (*p* < 0.05), whereas groups sharing at least one common letter are not significantly different.

**Figure 2:**
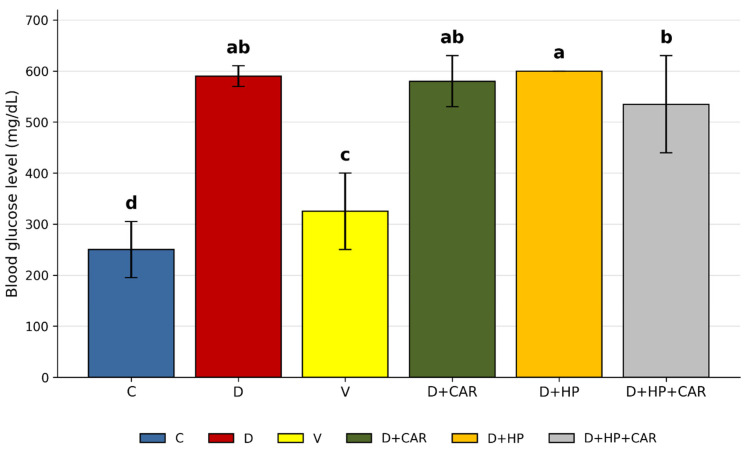
Blood glucose levels in the Control (C), Diabetic (D), Vehicle (V), Diabetic + Carvacrol (D + CAR), Diabetic + Hesperidin (D + HP), and Diabetic + Hesperidin + Carvacrol (D + HP + CAR) groups. Data are presented as mean ± standard deviation (SD). Different letters above the bars indicate statistically significant differences among groups (*p* < 0.05), whereas groups sharing at least one common letter are not significantly different.

**Figure 3:**
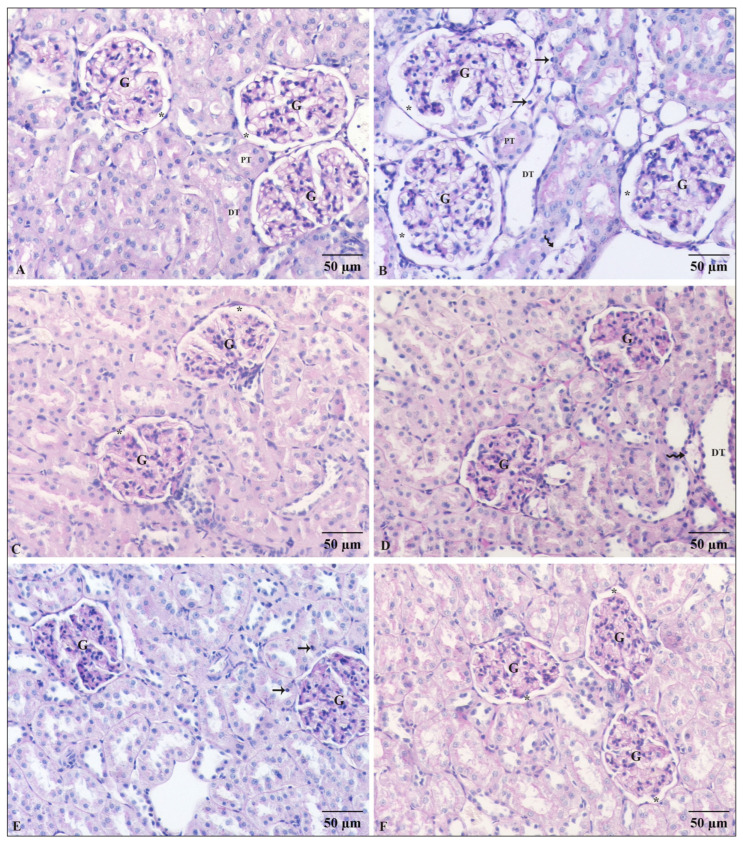
PAS-stained images of the renal cortex from different experimental groups. (**A**) The control group shows normal histological architecture, including glomeruli (G), proximal tubules (PT), distal tubules (DT), and Bowman’s space (asterisk). (**B**) The diabetic group displays glomeruli (G), proximal tubules (PT), dilated distal tubules (DT), Bowman’s space (asterisk), pyknotic nuclei (arrow), and tubules exhibiting ballooning degeneration (zigzag arrow). (**C**) The vehicle group exhibits histopathological features similar to the control group, Bowman’s space (asterisk). (**D**) The diabetic + carvacrol group shows dilated tubules (DT) and tubular epithelial cells undergoing ballooning degeneration (zigzag arrow). (**E**) The diabetic + hesperidin group exhibits pyknotic nuclei (arrows). (**F**) The diabetic + hesperidin + carvacrol group presents histopathological characteristics comparable to the control and vehicle groups, Bowman’s space (asterisk). PAS, ×200. Scale bar: 50 μm.

**Figure 4:**
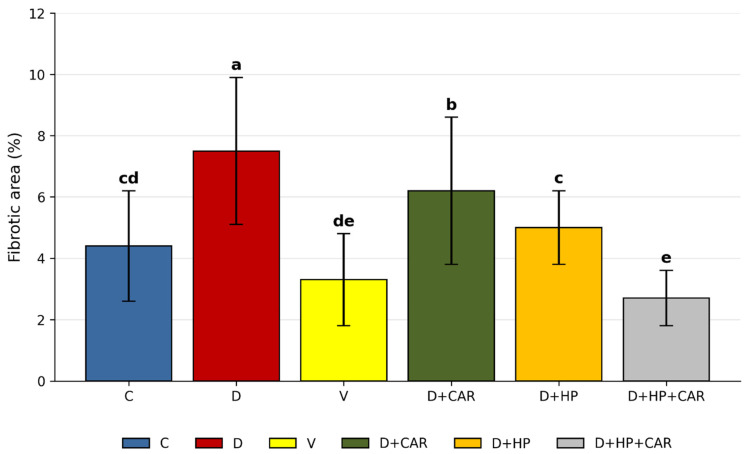
Fibrotic area (%) determined by Masson’s trichrome staining in the Control (C), Diabetic (D), Vehicle (V), Diabetic + Carvacrol (D + CAR), Diabetic + Hesperidin (D + HP), and Diabetic + Hesperidin + Carvacrol (D + HP + CAR) groups. Data are presented as mean ± standard deviation (SD). Different letters above the bars indicate statistically significant differences among groups (*p* < 0.05), whereas groups sharing at least one common letter are not significantly different.

**Figure 5:**
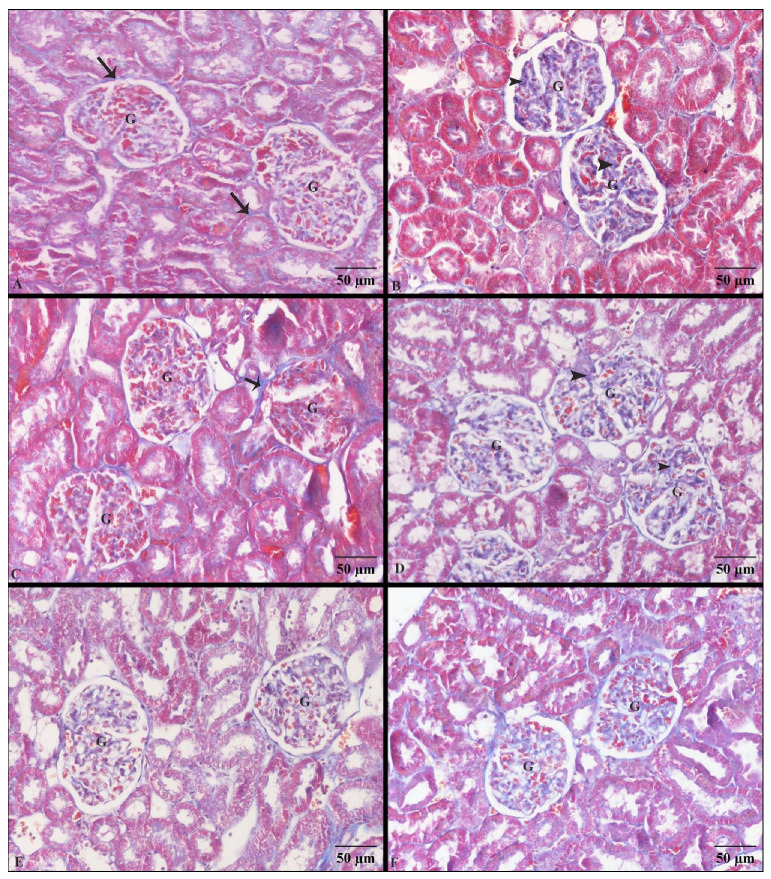
Masson’s trichrome stained sections showing collagen distribution in different experimental groups. (**A**) The control group demonstrates normal histological architecture, including glomeruli (G) and collagen deposition stained blue around normal glomeruli and within the interstitium (arrows). (**B**) The diabetic group shows more intensely stained collagen within the glomeruli (arrowhead). (**C**) The vehicle group displays histological features similar to the control group. (**D**) The diabetic + carvacrol group exhibits increased collagen deposition in the glomeruli (arrowhead). (**E**) The diabetic + hesperidin group shows collagen distribution patterns consistent with moderate structural changes. (**F**) The diabetic + hesperidin + carvacrol group demonstrates features comparable to the control and vehicle groups. Masson’s trichrome, ×200. Scale bar: 50 µm.

**Figure 6:**
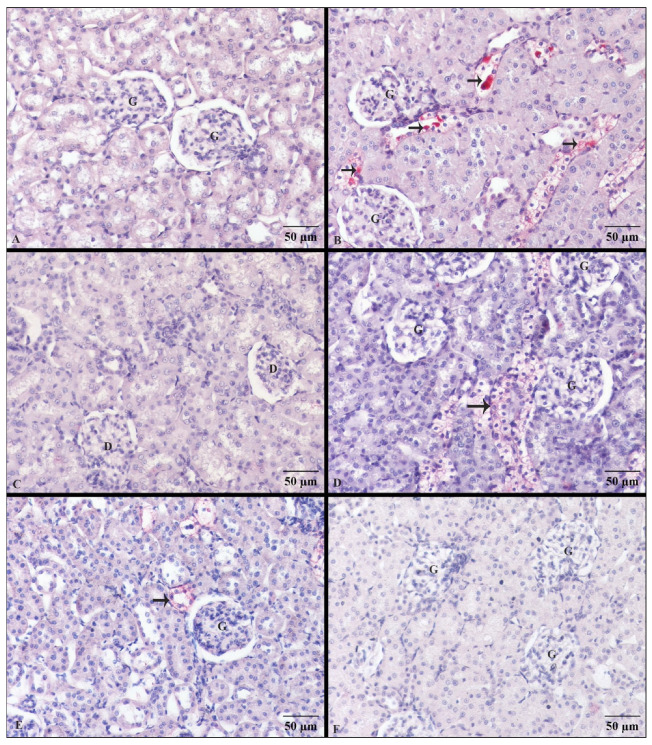
Best Carmine-stained cortical sections showing glycogen accumulation in different experimental groups. (**A**) The control group displays normal histological architecture, including glomeruli (G). (**B**) The diabetic group exhibits glycogen accumulation in the tubules (arrows). (**C**) The vehicle group shows histological features similar to the control group. (**D**) The diabetic + carvacrol group demonstrates mild glycogen accumulation in damaged tubules (arrows). (**E**) The diabetic + hesperidin group shows mild glycogen accumulation in damaged tubules (arrows). (**F**) The diabetic + hesperidin + carvacrol group presents histological characteristics comparable to the control group. Best Carmine, ×200. Scale bar: 50 µm.

**Figure 7:**
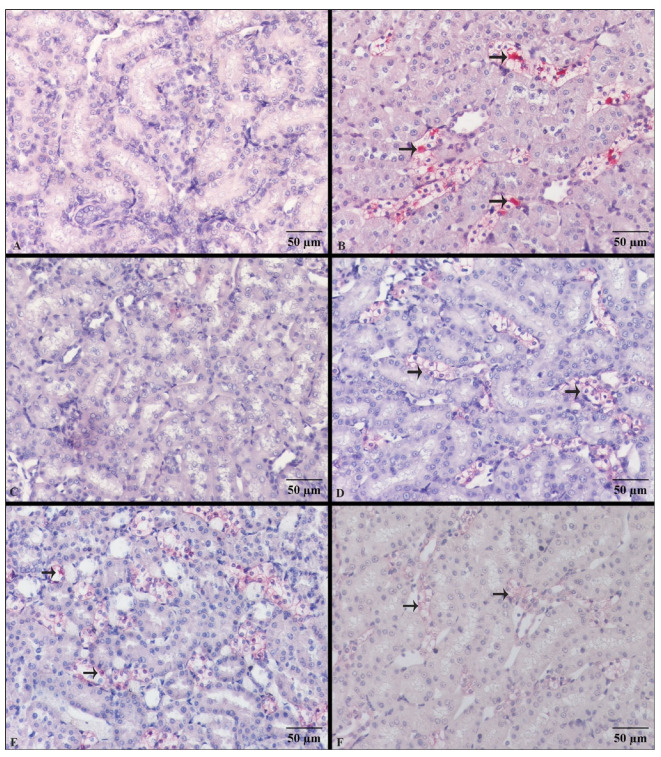
Best Carmine-stained medullary sections showing glycogen accumulation in different experimental groups. (**A**) The control group displays normal histological architecture. (**B**) The diabetic group exhibits glycogen accumulation in the tubules (arrows). (**C**) The vehicle group shows histological features comparable to the control group. (**D**) The diabetic + carvacrol group demonstrates mild glycogen accumulation in damaged tubules (arrows). (**E**) The diabetic + hesperidin group shows mild glycogen accumulation in damaged tubules (arrows). (**F**) The diabetic + hesperidin + carvacrol group exhibits mild glycogen accumulation in damaged tubules (arrows). Best Carmine, ×200. Scale bar: 50 µm.

**Figure 8:**
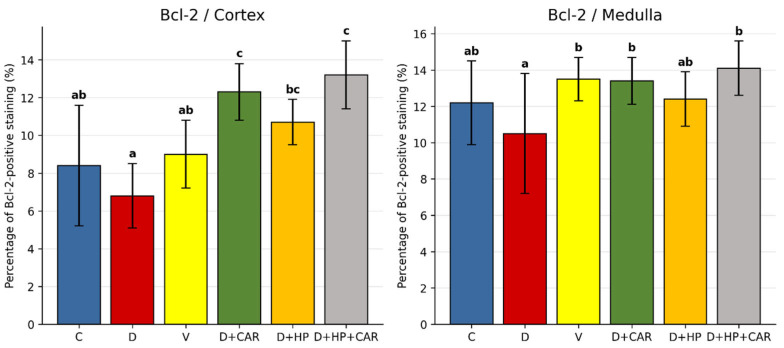
Percentage of Bcl-2-positive staining (%) in the renal cortex and medulla of the Control (C), Diabetic (D), Vehicle (V), Diabetic + Carvacrol (D + CAR), Diabetic + Hesperidin (D + HP), and Diabetic + Hesperidin + Carvacrol (D + HP + CAR) groups. Data are presented as mean ± standard deviation (SD). Different letters above the bars indicate statistically significant differences among groups (*p* < 0.05), whereas groups sharing at least one common letter are not significantly different.

**Figure 9:**
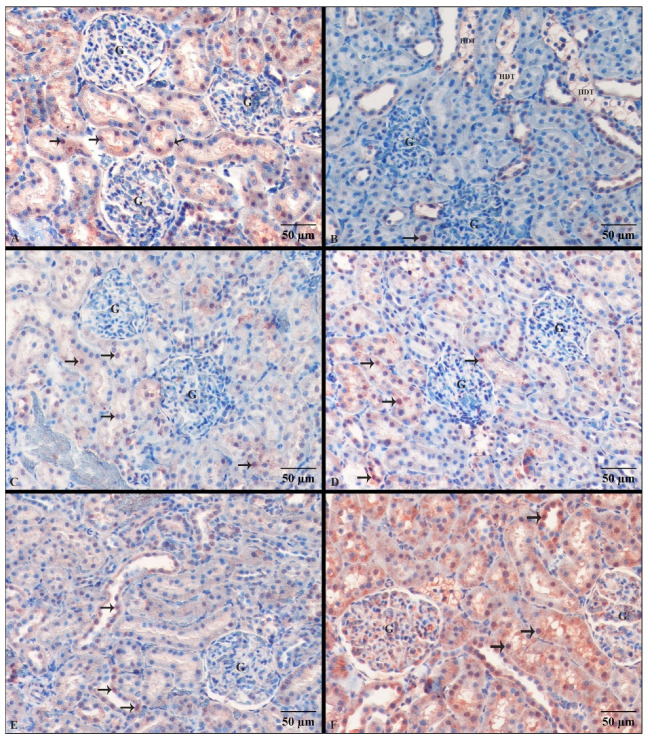
Bcl-2 immunohistochemical staining in the cortical region of different experimental groups. (**A**) The control group shows glomeruli (G) and tubular cells exhibiting cytoplasmic and nuclear immunopositivity (arrows). (**B**) The diabetic group displays glomeruli (G), damaged distal tubules (HDT), and a tubular cell with nuclear immunopositivity (arrow). (**C**) The vehicle group presents glomeruli (G) and tubular cells with nuclear immunopositivity (arrows). (**D**) The diabetic + carvacrol group shows glomeruli (G) and tubular cells demonstrating both cytoplasmic and nuclear immunopositivity (arrows). (**E**) The diabetic + hesperidin group exhibits glomeruli (G) and tubular cells with cytoplasmic and nuclear immunopositivity (arrows). (**F**) The diabetic + hesperidin + carvacrol group presents intense Bcl-2 immunopositivity, with glomeruli (G) and tubular cells showing cytoplasmic and nuclear immunopositivity (arrows). Bcl-2, ×200. Scale bar: 50 µm.

**Figure 10:**
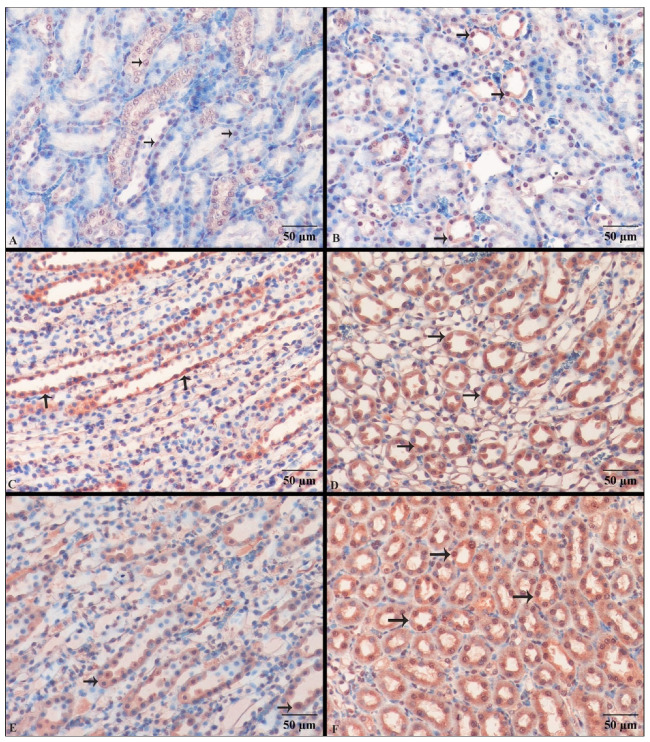
Bcl-2 immunohistochemical staining in the medullary region of different experimental groups. (**A**) Control group showing tubular cells with nuclear immunopositivity (arrows). (**B**) Diabetic group displaying tubular cells with both cytoplasmic and nuclear immunopositivity (arrows). (**C**) Vehicle group presenting tubular cells with cytoplasmic immunopositivity (arrows). (**D**) Diabetic + carvacrol group showing tubular cells exhibiting both cytoplasmic and nuclear immunopositivity (arrows). (**E**) Diabetic + hesperidin group showing tubular cells with cytoplasmic and nuclear immunopositivity (arrows). (**F**) Diabetic + hesperidin + carvacrol group exhibiting intense cytoplasmic and nuclear immunopositivity in tubular cells (arrows). Bcl-2, ×200. Scale bar: 50 µm.

**Figure 11:**
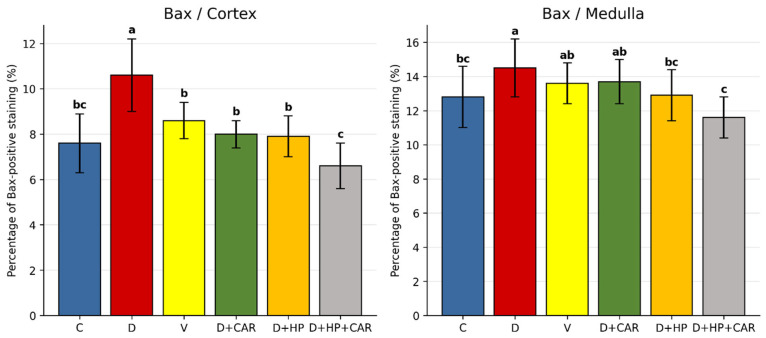
Percentage of Bax-positive staining (%) in the renal cortex and medulla of the Control (C), Diabetic (D), Vehicle (V), Diabetic + Carvacrol (D + CAR), Diabetic + Hesperidin (D + HP), and Diabetic + Hesperidin + Carvacrol (D + HP + CAR) groups. Data are presented as mean ± standard deviation (SD). Different letters above the bars indicate statistically significant differences among groups (*p* < 0.05), whereas groups sharing at least one common letter are not significantly different.

**Figure 12:**
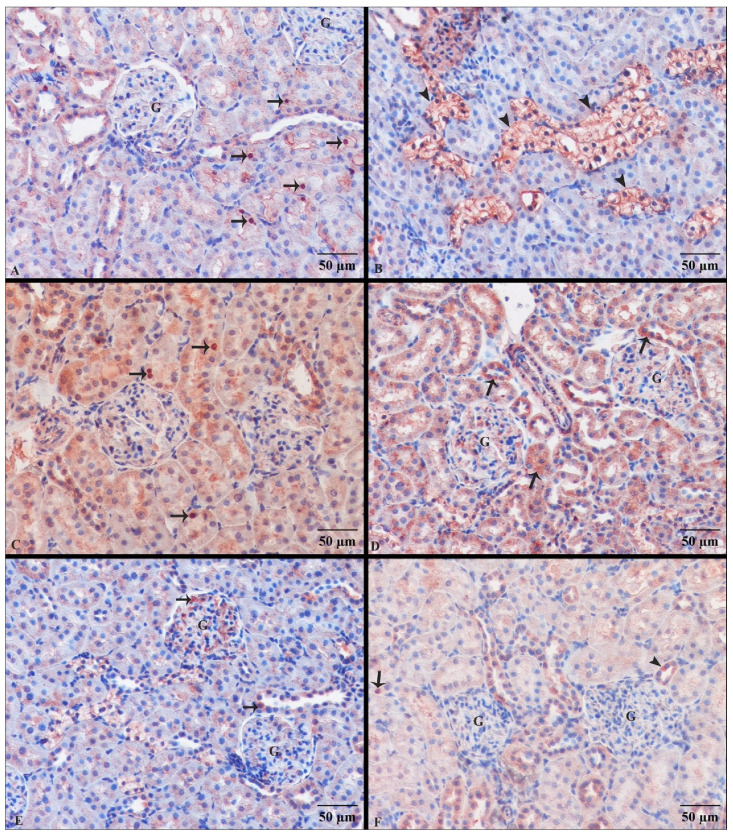
Bax immunohistochemical staining in the cortical region of different experimental groups. (**A**) Control group showing glomeruli (G) and tubular cells with nuclear immunopositivity (arrows). (**B**) Diabetic group displaying damaged tubules exhibiting cytoplasmic immunopositivity (arrowheads). (**C**) Vehicle group presenting tubular cells with nuclear immunopositivity (arrows). (**D**) Diabetic + carvacrol group showing tubular cells with cytoplasmic immunopositivity (arrows). (**E**) Diabetic + hesperidin group exhibiting tubular cells with cytoplasmic immunopositivity (arrows). (**F**) Diabetic + hesperidin + carvacrol group showing tubular cells with cytoplasmic (arrowhead) and nuclear (arrow) immunopositivity. Bax, ×200. Scale bar: 50 µm.

**Figure 13:**
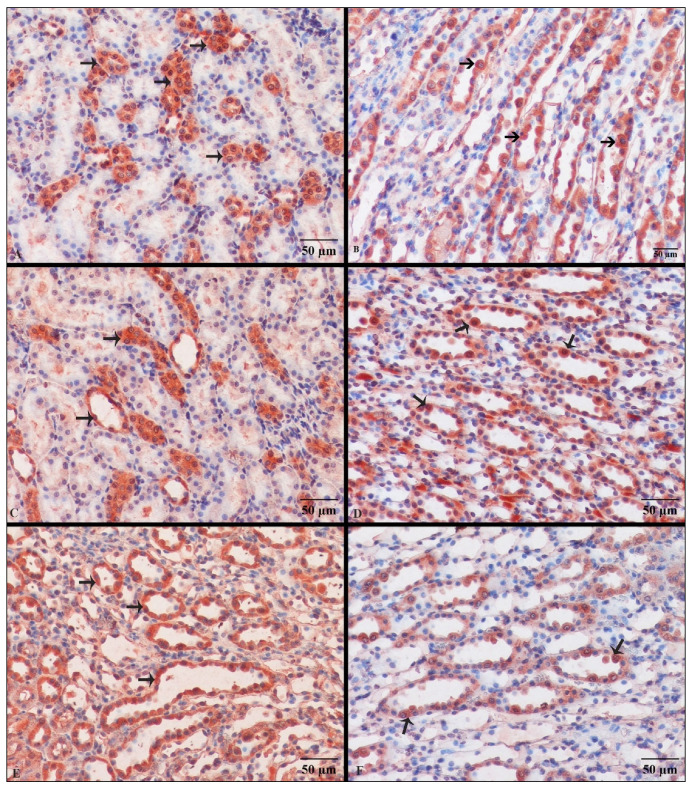
Bax immunohistochemical staining in the medullary region of different experimental groups. (**A**) Control group showing tubular cells with both cytoplasmic and nuclear immunopositivity (arrows). (**B**) Diabetic group displaying tubular cells with cytoplasmic immunopositivity (arrows). (**C**) Vehicle group presenting tubular cells with cytoplasmic and nuclear immunopositivity (arrows). (**D**) Diabetic + carvacrol group showing tubular cells exhibiting both cytoplasmic and nuclear immunopositivity (arrows). (**E**) Diabetic + hesperidin group displaying tubular cells with cytoplasmic immunopositivity (arrows). (**F**) Diabetic + hesperidin + carvacrol group showing tubular cells with both cytoplasmic and nuclear immunopositivity (arrows). Bax, ×200. Scale bar: 50 µm.

**Figure 14:**
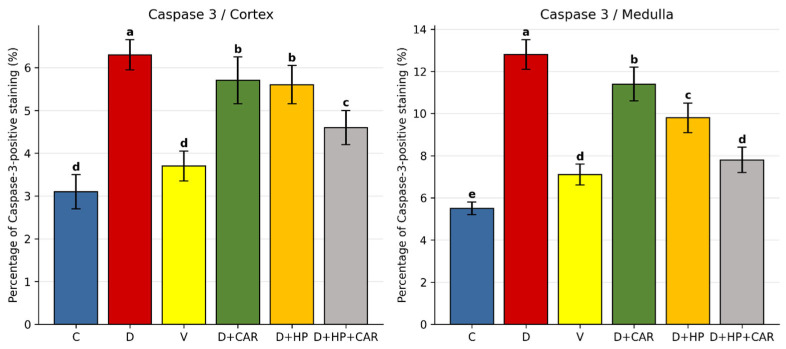
Percentage of Caspase-3-positive staining (%) in the renal cortex and medulla of the Control (C), Diabetic (D), Vehicle (V), Diabetic + Carvacrol (D + CAR), Diabetic + Hesperidin (D + HP), and Diabetic + Hesperidin + Carvacrol (D + HP + CAR) groups. Data are presented as mean ± standard deviation (SD). Different letters above the bars indicate statistically significant differences among groups (*p* < 0.05), whereas groups sharing at least one common letter are not significantly different.

**Figure 15:**
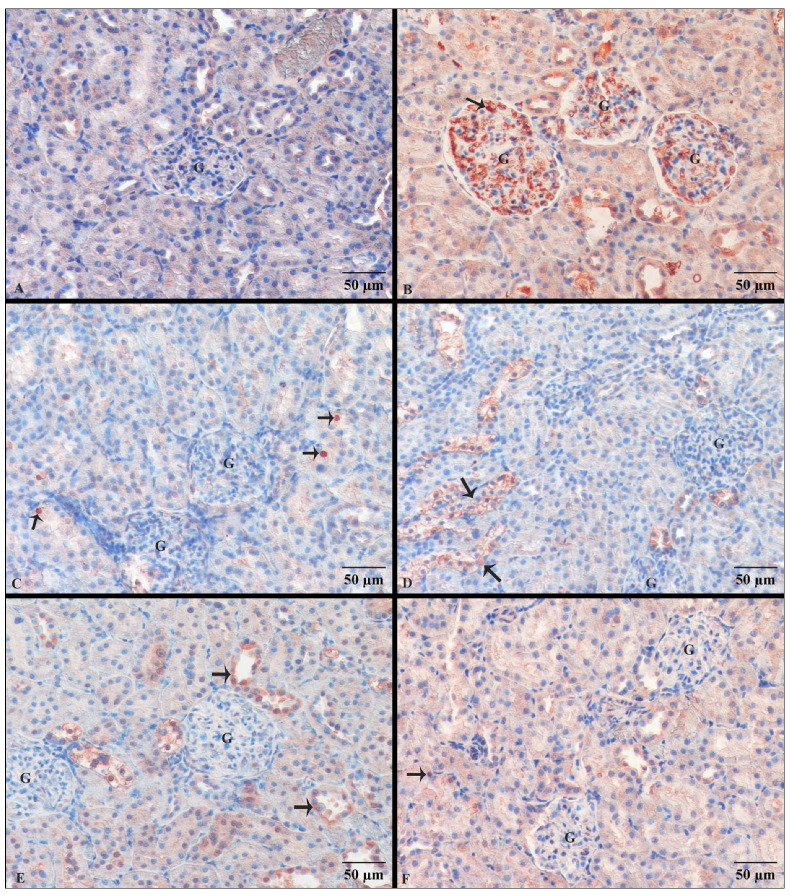
Active caspase-3 immunohistochemical staining in the cortical region of different experimental groups. (**A**) Control group showing normal glomerular structure (G). (**B**) Diabetic group displaying glomerular cells with cytoplasmic immunopositivity (arrows). (**C**) Vehicle group presenting tubular cells with nuclear immunopositivity (arrows). (**D**) Diabetic + carvacrol group showing tubular cells with cytoplasmic immunopositivity (arrows). (**E**) Diabetic + hesperidin group exhibiting tubular cells with cytoplasmic immunopositivity (arrows). (**F**) Diabetic + hesperidin + carvacrol group showing tubular cells with nuclear immunopositivity (arrows). Active caspase-3, ×200. Scale bar: 50 µm.

**Figure 16:**
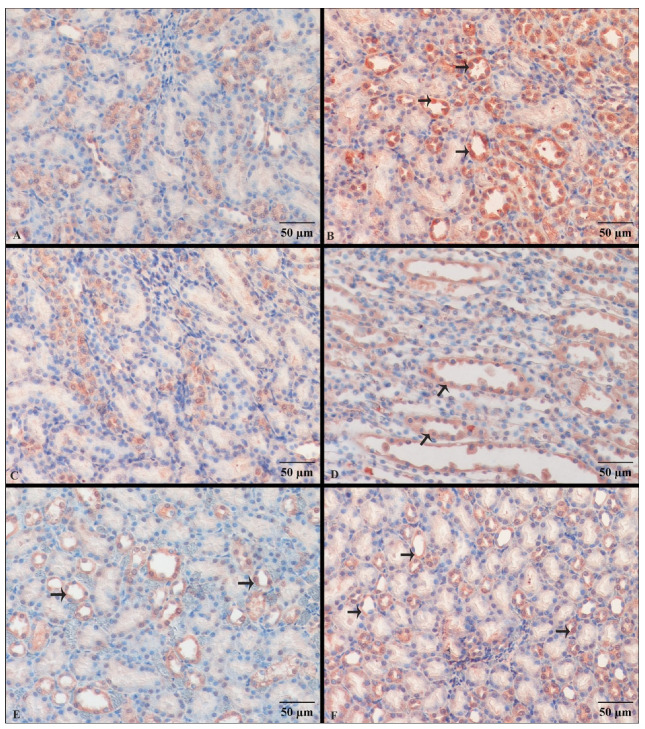
Active caspase-3 immunohistochemical staining in the medullary region of different experimental groups. (**A**) Control group showing normal medullary morphology. (**B**) Diabetic group displaying tubular cells with cytoplasmic immunopositivity (arrows). (**C**) Vehicle group presenting histological features comparable to the control group. (**D**) Diabetic + carvacrol group showing tubular cells with cytoplasmic immunopositivity (arrows). (**E**) Diabetic + hesperidin group exhibiting tubular cells with cytoplasmic immunopositivity (arrows). (**F**) Diabetic + hesperidin + carvacrol group demonstrating tubular cells with both cytoplasmic and nuclear immunopositivity (arrows). Active caspase-3, ×200. Scale bar: 50 µm.

**Figure 17:**
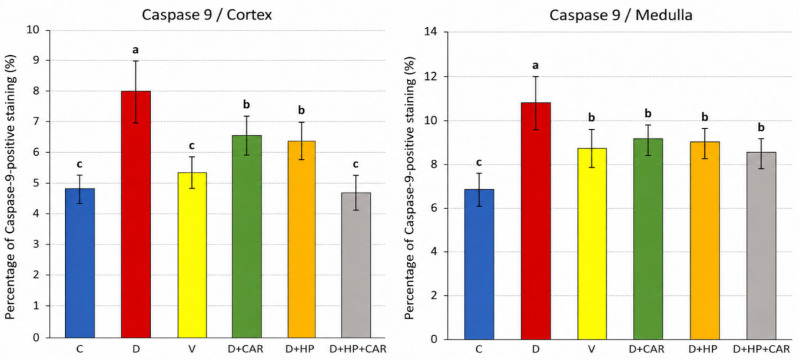
Percentage of Caspase-9-positive staining (%) in the renal cortex and medulla of the Control (C), Diabetic (D), Vehicle (V), Diabetic + Carvacrol (D + CAR), Diabetic + Hesperidin (D + HP), and Diabetic + Hesperidin + Carvacrol (D + HP + CAR) groups. Data are presented as mean ± standard deviation (SD). Different letters above the bars indicate statistically significant differences among groups (*p* < 0.05), whereas groups sharing at least one common letter are not significantly different.

**Figure 18:**
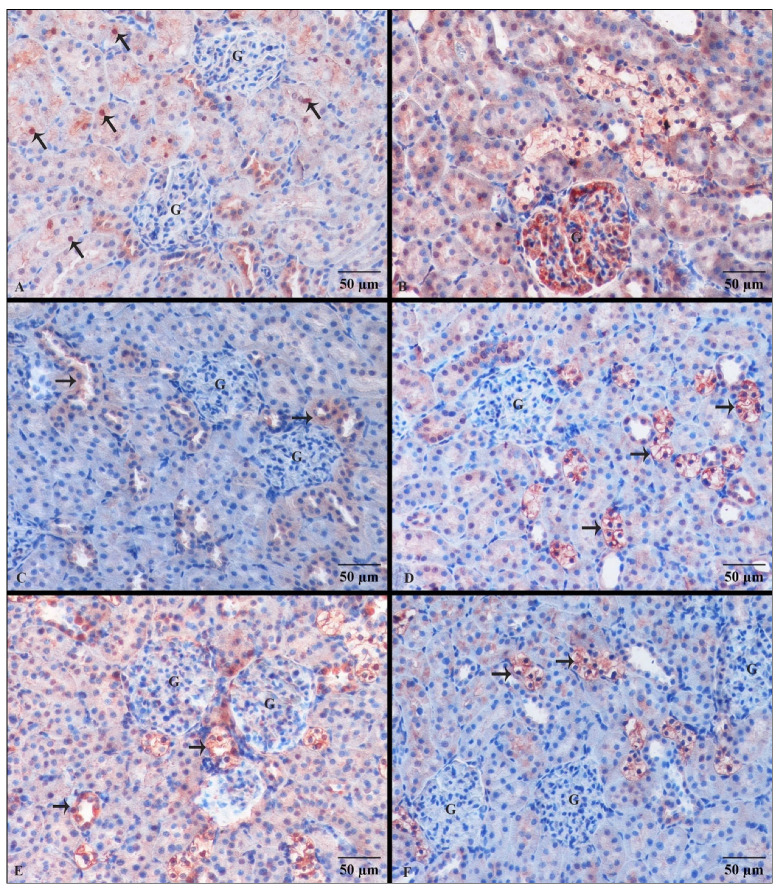
Caspase-9 immunohistochemical staining in the cortical region of different experimental groups. (**A**) Control group showing glomeruli (G) and tubular cells with nuclear immunopositivity (arrows). (**B**) Diabetic group displaying glomeruli (G) with intense cytoplasmic and nuclear immunopositivity. (**C**) Vehicle group presenting tubular cells with cytoplasmic immunopositivity (arrows). (**D**) Diabetic + carvacrol group showing tubular cells with cytoplasmic immunopositivity (arrows). (**E**) Diabetic + hesperidin group exhibiting tubular cells with cytoplasmic immunopositivity (arrows). (**F**) Diabetic + hesperidin + carvacrol group demonstrating tubular cells with cytoplasmic immunopositivity (arrows). Caspase-9, ×200. Scale bar: 50 µm.

**Figure 19:**
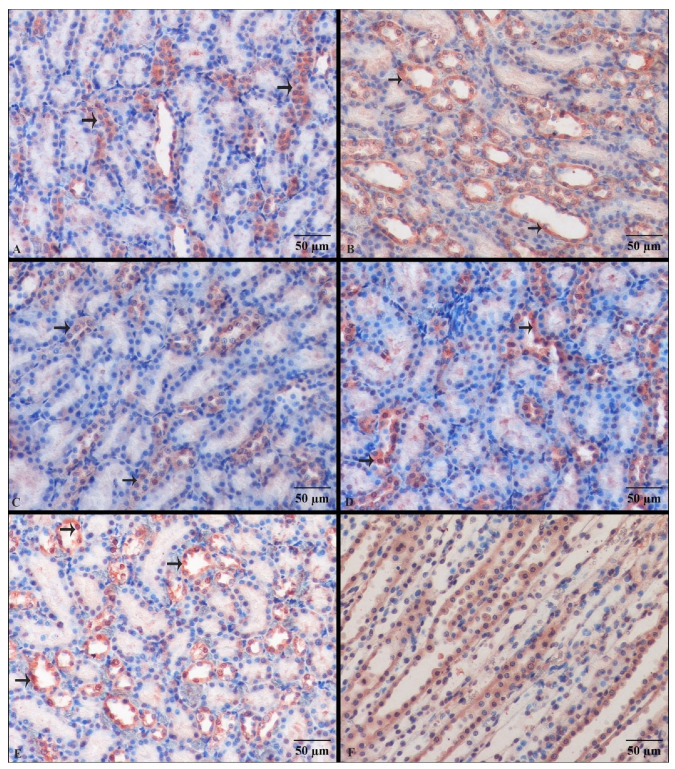
Caspase-9 immunohistochemical staining in the medullary region of different experimental groups. (**A**) Control group showing tubular cells with cytoplasmic immunopositivity (arrows). (**B**) Diabetic group displaying tubular cells with cytoplasmic immunopositivity (arrows). (**C**) Vehicle group presenting tubular cells with both cytoplasmic and nuclear immunopositivity (arrows). (**D**) Diabetic + carvacrol group showing tubular cells exhibiting cytoplasmic and nuclear immunopositivity (arrows). (**E**) Diabetic + hesperidin group displaying tubular cells with cytoplasmic and nuclear immunopositivity (arrows). (**F**) Diabetic + hesperidin + carvacrol group showing tubular cells with cytoplasmic and nuclear immunopositivity. Caspase-9, ×200. Scale bar: 50 µm.

**Figure 20:**
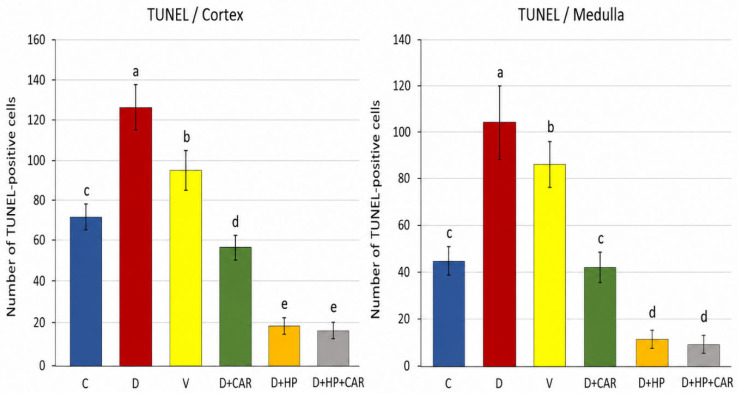
Number of TUNEL-positive cells in the renal cortical and medullary regions of the Control (C), Diabetic (D), Vehicle (V), Diabetic + Carvacrol (D + CAR), Diabetic + Hesperidin (D + HP), and Diabetic + Hesperidin + Carvacrol (D + HP + CAR) groups. Data are presented as mean ± standard deviation (SD). Different letters above the bars indicate statistically significant differences among groups (*p* < 0.05), whereas groups sharing at least one common letter are not significantly different.

**Figure 21:**
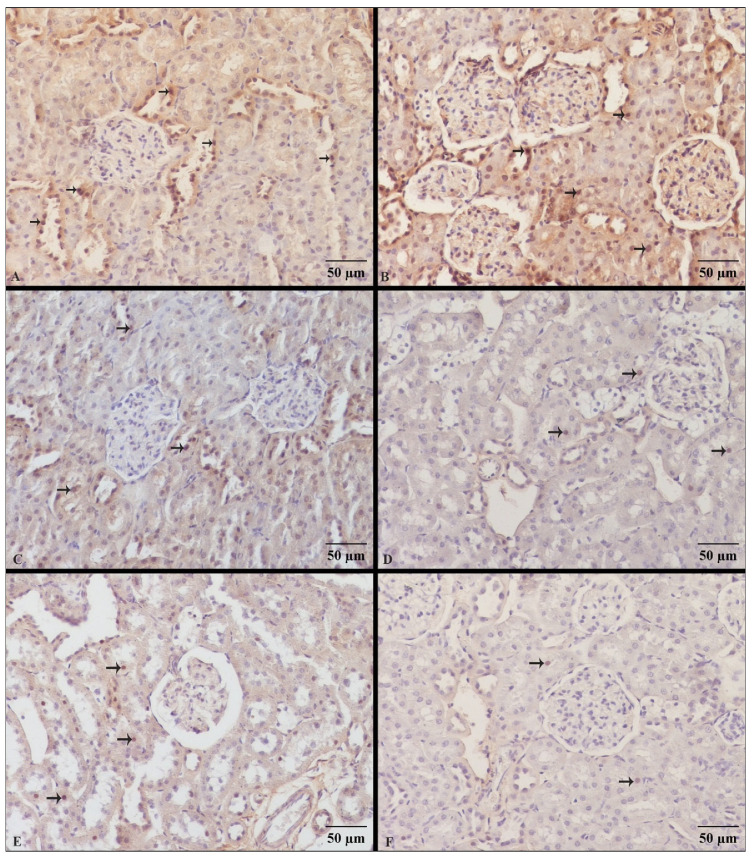
TUNEL staining in the cortical region of different experimental groups. (**A**) Control group showing tubular cells with TUNEL positivity (arrows). (**B**) Diabetic group displaying tubular cells with TUNEL positivity (arrows). (**C**) Vehicle group presenting tubular cells with TUNEL positivity (arrows). (**D**) Diabetic + carvacrol group showing tubular cells exhibiting TUNEL positivity (arrows). (**E**) Diabetic + hesperidin group displaying tubular cells with TUNEL positivity (arrows). (**F**) Diabetic + hesperidin + carvacrol group showing tubular cells with TUNEL positivity (arrows). TUNEL, ×200. Scale bar: 50 µm.

**Figure 22:**
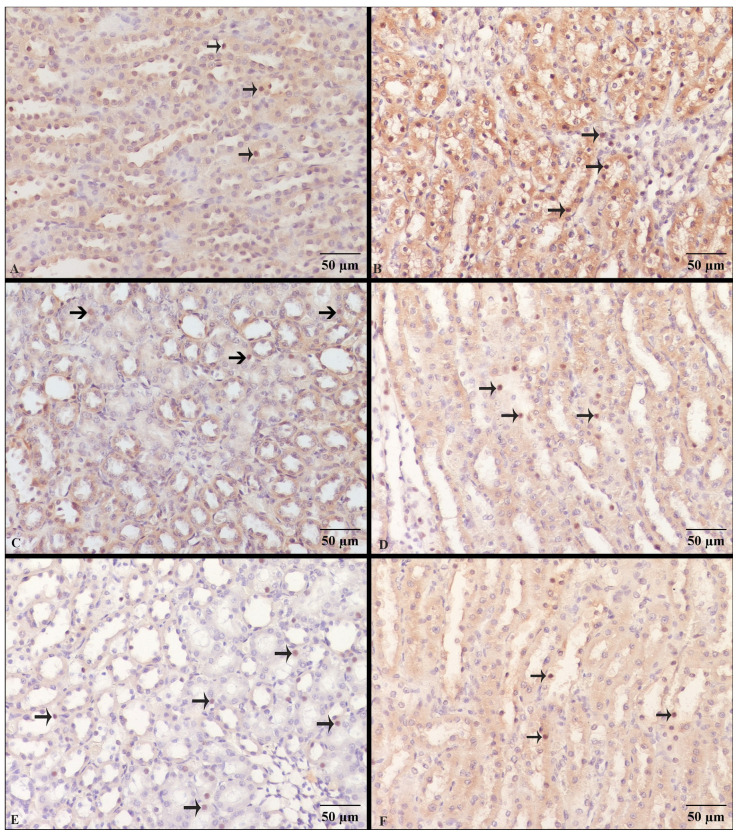
TUNEL staining in the medullary region of different experimental groups. (**A**) Control group showing tubular cells with TUNEL positivity (arrows). (**B**) Diabetic group displaying tubular cells with TUNEL positivity (arrows). (**C**) Vehicle group presenting tubular cells with TUNEL positivity (arrows). (**D**) Diabetic + carvacrol group showing tubular cells exhibiting TUNEL positivity (arrows). (**E**) Diabetic + hesperidin group displaying tubular cells with TUNEL positivity (arrows). (**F**) Diabetic + hesperidin + carvacrol group showing tubular cells with TUNEL positivity (arrows). TUNEL, ×200. Scale bar: 50 µm.

**Table 1:** Changes in body weight of experimental groups during the study period. Body weights of rats were recorded on day 1 and weekly throughout the experimental period. Additional measurements were obtained during the metabolic cage period and at necropsy. Data are presented as mean ± standard deviation (SD). Different superscript letters within the same column indicate statistically significant differences among groups (*p* < 0.05). Groups sharing at least one common letter are not significantly different. C: Control, D: Diabetic, V: Vehicle, D + CAR: Diabetic + carvacrol, D + HP: Diabetic + hesperidin, D + HP + CAR: Diabetic + hesperidin + carvacrol.

Groups	1. Day (g)	1. Week (g)	2. Week (g)	3. Week (g)	4. Week (g)	5. Week (g)	6. Week (g)	Metabolic Cage (g)	Necropsy (g)
C	132.20 ± 12.45 ^a^	145.53 ± 14.74 ^a^	168.30 ± 16.07 ^a^	199.10 ± 12.07 ^a^	227.15 ± 19.53 ^a^	234.70 ± 22.36 ^a^	248.90 ± 24.74 ^a^	279.90 ± 23.77 ^a^	279.30 ± 30.00 ^a^
D	122.50 ± 8.95 ^a^	127.23 ± 9.65 ^b^	147.03 ± 11.92 ^b^	166.03 ± 15.63 ^b^	181.55 ± 15.86 ^b^	187.35 ± 20.12 ^b^	191.63 ± 24.79 ^b^	189.40 ± 24.82 ^b^	189.40 ± 24.82 ^b^
V	166.90 ± 9.81 ^bc^	180.88 ± 10.61 ^d^	204.26 ± 12.40 ^d^	222.51 ± 14.00 ^d^	236.93 ± 14.08 ^a^	255.20 ± 13.48 ^c^	272.51 ± 13.72 ^c^	283.00 ± 14.98 ^a^	284.70 ± 13.54 ^a^
D + CAR	152.89 ± 12.58 ^ab^	147.30 ± 9.72 ^a^	153.51 ± 8.21 ^b^	164.73 ± 10.20 ^b^	165.79 ± 14.31 ^c^	174.41 ± 18.62 ^d^	172.96 ± 18.72 ^d^	174.67 ± 20.45 ^c^	175.33 ± 20.56 ^c^
D + HP	182.11 ± 31.47 ^c^	175.07 ± 33.59 ^cd^	166.00 ± 37.94 ^abc^	151.65 ± 19.35 ^b^	163.11 ± 21.23 ^c^	166.40 ± 18.14 ^d^	169.20 ± 21.83 ^d^	183.11 ± 29.45 ^bc^	180.89 ± 29.50 ^bc^
D + HP + CAR	161.10 ± 12.07 ^bc^	162.79 ± 12.20 ^c^	181.30 ± 16.26 ^c^	195.12 ± 18.33 ^a^	203.72 ± 20.44 ^b^	208.85 ± 29.03 ^b^	213.97 ± 30.44 ^b^	217.90 ± 30.35 ^b^	218.60 ± 28.89 ^b^

**Table 2:** Morphometric results of the right kidney in the experimental groups. Data are presented as mean ± standard deviation (SD). Different superscript letters within the same column indicate statistically significant differences (*p* < 0.05). Values sharing at least one common letter are not significantly different, whereas values with no common letters differ significantly. C: Control; D: Diabetic; V: Vehicle; D + CAR: Diabetic + carvacrol; D + HP: Diabetic + hesperidin; D + HP + CAR: Diabetic + hesperidin + carvacrol.

Groups	Kidney Weight (gr)	Length (mm)	Width (mm)	Cortex (mm)	Medulla (mm)
C	1.244 ± 0.07 ^b^	17.92 ± 0.6	7.6 ± 0.64	1.89 ± 0.31 ^b^	1.91 ± 0.56
D	1.137 ± 0.12 ^bc^	16.83 ± 1.65	7.44 ± 1.18	1.96 ± 0.5 ^ab^	1.56 ± 0.36
V	1.239 ± 0.08 ^b^	16.71 ± 0.55	7.53 ± 0.9	2.29 ± 0.46 ^ab^	1.8 ± 0.56
D + CAR	1.174 ± 0.15 ^bc^	17.33 ± 0.87	7.47 ± 1.32	2.17 ± 0.46 ^ab^	1.68 ± 0.25
D + HP	1.111 ± 0.14 ^c^	17.61 ± 0.9	7.6 ± 1.13	2.37 ± 0.36 ^a^	1.68 ± 0.34
D + HP + CAR	1.361 ± 0.16 ^a^	17.78 ± 1.07	7.5 ± 1.18	1.9 ± 0.34 ^b^	1.41 ± 0.18
*p*	**0.001**	0.059	0.999	**0.047**	0.149

**Table 3:** Morphometric results of the left kidney in the experimental groups. Data are presented as mean ± standard deviation (SD). Different superscript letters within the same column indicate statistically significant differences (*p* < 0.05). Values sharing at least one common letter are not significantly different, whereas values with no common letters differ significantly. C: Control; D: Diabetic; V: Vehicle; D + CAR: Diabetic + carvacrol; D + HP: Diabetic + hesperidin; D + HP + CAR: Diabetic + hesperidin + carvacrol.

Groups	Kidney Weight (gr)	Length (mm)	Width (mm)	Cortex (mm)	Medulla (mm)
C	1.33 ± 0.23 ^a^	17.92 ± 0.6	7.53 ± 0.75	1.99 ± 0.49	1.73 ± 0.29
D	1.089 ± 0.14 ^c^	18.76 ± 0.99	6.91 ± 0.64	2.1 ± 0.45	1.92 ± 0.54
V	1.183 ± 0.11 ^bc^	17.91 ± 0.98	7.73 ± 0.84	2.25 ± 0.47	1.69 ± 0.33
D + CAR	1.094 ± 0.1 ^c^	17.93 ± 0.9	7.3 ± 1.51	1.73 ± 0.35	1.43 ± 0.33
D + HP	1.116 ± 0.12 ^c^	17.99 ± 1.03	7.59 ± 0.75	1.84 ± 0.53	1.58 ± 0.45
D + HP + CAR	1.295 ± 0.15 ^ab^	18.62 ± 1.75	7.27 ± 1.66	1.9 ± 0.42	1.5 ± 0.4
*p*	**0.001**	0.338	0.652	0.189	0.138

**Table 4:** Relative weights of the right and left kidneys in the experimental groups. Data are presented as mean ± standard deviation (SD). Different superscript letters within the same column indicate statistically significant differences (*p* < 0.05). Values sharing at least one common letter are not significantly different, whereas values with no common letters differ significantly. C: Control; D: Diabetic; V: Vehicle; D + CAR: Diabetic + carvacrol; D + HP: Diabetic + hesperidin; D + HP + CAR: Diabetic + hesperidin + carvacrol.

Groups	Right Kidney Weight (gr)	Left Kidney Weight (gr)
C	0.44 ± 0.04 ^a^	0.47 ± 0.06 ^a^
D	0.60 ± 0.03 ^b^	0.57 ± 0.05 ^c^
V	0.43 ± 0.01 ^a^	0.41 ± 0.02 ^b^
D + CAR	0.67 ± 0.09 ^c^	0.62 ± 0.03 ^c^
D + HP	0.61 ± 0.06 ^bc^	0.62 ± 0.08 ^c^
D + HP + CAR	0.62 ± 0.08 ^bc^	0.59 ± 0.05 ^c^
*p*	**0.001**	**0.001**

**Table 5:** Glomerular size, Bowman’s space width, visceral layer of Bowman’s capsule (BKv), parietal layer of Bowman’s capsule (BKp), and tubular basement membrane (TBM) thickness in the experimental groups. Data are presented as mean ± standard deviation (SD). Different superscript letters within the same column indicate statistically significant differences (*p* < 0.05). Values sharing at least one common letter are not significantly different, whereas values with no common letters differ significantly. C: Control; D: Diabetic; V: Vehicle; D + CAR: Diabetic + carvacrol; D + HP: Diabetic + hesperidin; D + HP + CAR: Diabetic + hesperidin + carvacrol. BKv: Visceral layer of Bowman’s capsule; BKp: Parietal layer of Bowman’s capsule; TBM: Tubular basement membrane.

Groups	Glomerular Size (µm)	Bowman’s Space Width (µm)	BKv (µm)	BKp (µm)	TBM (µm)
C	94.22 ± 6.1 ^ab^	11.34 ± 3	0.98 ± 0.2	1.79 ± 0.2 ^a^	0.72 ± 0.1 ^a^
D	104.04 ± 7.3 ^a^	7.53 ± 2.3	0.95 ± 0.1	1.43 ± 0.1 ^b^	0.65 ± 0.1 ^ab^
V	82.5 ± 15.9 ^b^	10.28 ± 1.9	1.01 ± 0.2	1.48 ± 0.2 ^b^	0.62 ± 0.1 ^ab^
D + CAR	87.73 ± 6.6 ^b^	10.34 ± 1.8	0.75 ± 0	1.32 ± 0.2 ^b^	0.71 ± 0 ^a^
D + HP	86.27 ± 6.4 ^b^	10.57 ± 2.4	0.87 ± 0.2	1.36 ± 0.1 ^b^	0.57 ± 0.1 ^b^
D + HP + CAR	89.6 ± 8.6 ^b^	10.87 ± 2.3	0.87 ± 0.1	1.38 ± 0.1 ^b^	0.62 ± 0 ^ab^
*p*	**0.017**	0.185	0.247	**0.001**	**0.03**

**Table 6:** Tubular dilatation (TD), tubular atrophy (TA), intracellular vacuolization (ISV), hyaline accumulation (HA), mononuclear cell infiltration (MCI), number of sclerotic glomeruli (NSG), and mesangial matrix expansion (MM) in the experimental groups. Data are presented as median (minimum–maximum). Statistical analysis was performed using the Kruskal–Wallis test followed by Dunn’s multiple comparison test. Different superscript letters within the same column indicate statistically significant differences (*p* < 0.05). Values sharing at least one common letter are not significantly different, whereas values with no common letters differ significantly. C: Control; D: Diabetic; V: Vehicle; D + CAR: Diabetic + carvacrol; D + HP: Diabetic + hesperidin; D + HP + CAR: Diabetic + hesperidin + carvacrol. TD: Tubular dilatation; TA: Tubular atrophy; ISV: Intracellular vacuolization; HA: Hyaline accumulation; MCI: Mononuclear cell infiltration; NSG: Number of sclerotic glomeruli; MM: Mesangial matrix expansion.

Groups	TD	TA	ISV	HB	MHİ	SGS	MMA
C	0 (0:0) ^b^	0 (0:0)	0 (0:0) ^b^	0 (0:0)	0 (0:0)	2 (1:3)	0 (0:1) ^e^
D	1 (1:1) ^a^	0 (0:0)	2 (0:2) ^a^	0 (0:0)	0 (0:0)	5 (4:7)	3 (2:3) ^a^
V	0 (0:0) ^b^	0 (0:0)	0 (0:0) ^b^	0 (0:0)	0 (0:0)	3 (2:5)	1 (0:1) ^de^
D + CAR	1 (0:1) ^ab^	0 (0:0)	1 (0:1) ^b^	0 (0:0)	0 (0:0)	4 (3:5)	1 (1:2) ^cd^
D + HP	1 (0:1) ^ab^	0 (0:0)	1 (0:1) ^b^	0 (0:0)	0 (0:0)	5 (1:7)	2 (2:2) ^b^
D + HP + CAR	0 (0:1) ^ab^	0 (0:0)	0 (0:1)	0 (0:0)	0 (0:0)	4 (2:6)	2 (1:2) ^bc^
*p*	**0.011**	1	**<0.001**	1	1	0.056	**<0.001**

## Data Availability

Data that support the findings of this study are available from the corresponding author upon reasonable request.

## Abbreviations

Apaf-1, Apoptotic protease activating factor-1; Bax, B-cell lymphoma 2-associated X protein; Bcl-2, B-cell lymphoma 2; BW, Body weight; CAR, Carvacrol; DAB, 3,3′-Diaminobenzidine; DM, Diabetes mellitus; DN, Diabetic nephropathy; DKD, Diabetic kidney disease; DMSO, Dimethyl sulfoxide; ER, Endoplasmic reticulum; FADD, Fas-associated death domain; HP, Hesperidin; HRP, Horseradish peroxidase; IHC, Immunohistochemistry; IP, Intraperitoneal; MAC, Mitochondria-associated channel; MOMP, Mitochondrial outer membrane permeabilization; PAS, Periodic Acid–Schiff; PBS, Phosphate-buffered saline; PTPC, Permeability transition pore complex; RAS, Renin–angiotensin system; ROS, Reactive oxygen species; RT-PCR, Real-Time Polymerase Chain Reaction; SBPC, Streptavidin–Biotin Peroxidase Complex; STZ, Streptozotocin; TdT, Terminal deoxynucleotidyl transferase; TUNEL, Terminal deoxynucleotidyl transferase dUTP nick-end labeling.
